# Maternal exposure to high‐fat diet during pregnancy and lactation predisposes normal weight offspring mice to develop hepatic inflammation and insulin resistance

**DOI:** 10.14814/phy2.14811

**Published:** 2021-03-26

**Authors:** Suchaorn Saengnipanthkul, Hye Lim Noh, Randall H. Friedline, Sujin Suk, Stephanie Choi, Nicholas K. Acosta, Duy A. Tran, Xiaodi Hu, Kunikazu Inashima, Allison M. Kim, Ki Won Lee, Jason K. Kim

**Affiliations:** ^1^ Division of Nutrition Department of Pediatrics Faculty of Medicine Khon Kaen University Khon Kaen Thailand; ^2^ Program in Molecular Medicine University of Massachusetts Medical School Worcester MA USA; ^3^ Division of Endocrinology, Metabolism, and Diabetes Department of Medicine University of Massachusetts Medical School Worcester MA USA; ^4^ Department of Agricultural Biotechnology College of Agricultural and Life Sciences Seoul National University Seoul South Korea

**Keywords:** high‐fat diet, inflammation, insulin resistance, lactation, liver, maternal nutrition

## Abstract

Increasing evidence shows a potential link between the perinatal nutrient environment and metabolic outcome in offspring. Here, we investigated the effects of maternal feeding of a high‐fat diet (HFD) during the perinatal period on hepatic metabolism and inflammation in male offspring mice at weaning and in early adulthood. Female C57BL/6 J mice were fed HFD or normal chow (NC) for 4 weeks before mating and during pregnancy and lactation. The male offspring mice were weaned onto an NC diet, and metabolic and molecular experiments were performed in early adulthood. At postnatal day 21, male offspring mice from HFD‐fed dams (Off‐HFD) showed significant increases in whole body fat mass and fasting levels of glucose, insulin, and cholesterol compared to male offspring mice from NC‐fed dams (Off‐NC). The RT‐qPCR analysis showed two‐ to fivefold increases in hepatic inflammatory markers (MCP‐1, IL‐1β, and F4/80) in Off‐HFD mice. Hepatic expression of G6Pase and PEPCK was elevated by fivefold in the Off‐HFD mice compared to the Off‐NC mice. Hepatic expression of GLUT4, IRS‐1, and PDK4, as well as lipid metabolic genes, CD36, SREBP1c, and SCD1 were increased in the Off‐HFD mice compared to the Off‐NC mice. In contrast, CPT1a mRNA levels were reduced by 60% in the Off‐HFD mice. At postnatal day 70, despite comparable body weights to the Off‐NC mice, Off‐HFD mice developed hepatic inflammation with increased expression of MCP‐1, CD68, F4/80, and CD36 compared to the Off‐NC mice. Despite normal body weight, Off‐HFD mice developed insulin resistance with defects in hepatic insulin action and insulin‐stimulated glucose uptake in skeletal muscle and brown fat, and these metabolic effects were associated with hepatic inflammation in Off‐HFD mice. Our findings indicate hidden, lasting effects of maternal exposure to HFD during pregnancy and lactation on metabolic homeostasis of normal weight offspring mice.

## Graphical abstract

We investigated the effects of maternal feeding of a high‐fat diet (HFD) during the perinatal period on hepatic metabolism and inflammation in male offspring mice at weaning and in early adulthood. Offspring from HFD showed transient increase in adiposity, fasting blood glucose, and cholesterol levels at weaning. Young adult male mice developed systemic insulin resistance with defects in hepatic insulin action and insulin‐stimulated glucose uptake in skeletal muscle and brown adipose tissue. Our findings indicate hidden, lasting effects of maternal exposure to HFD on metabolic homeostasis of normal weight offspring mice.

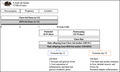

**Highlights**

- Maternal consumption of a high‐fat diet during pregnancy and lactation caused a transient increase in adiposity, fasting blood glucose, insulin, and cholesterol levels at weaning.
- Male offsping mice from HFD‐fed mothers developed hepatic inflammation with upregulation of proinflammatory genes and macrophages and increased glucose metabolic genes in early postnatal period.
- Young adult male mice developed systemic insulin resistance with defects in hepatic insulin action and insulin‐stimulated glucose uptake in skeletal muscle and brown adipose tissue.
- Despite normal body weight, there are hidden changes in hepatic inflammation and whole body glucose metabolism in offspring mice.

## 1 INTRODUCTION

Over the past decade, many studies have focused on the “developmental origins of health and disease (DOHaD)” hypothesis, which stimulated interest in the fetal origins of adult disorders (Barker, 2007). The prevalence of obesity has increased significantly over time due to progressive changes in dietary patterns and lifestyle. Recent studies have shown the effects of maternal obesity on the metabolic outcomes of offspring, including macrosomia, obesity, type 2 diabetes, hypertension and cardiovascular disease, and dyslipidemia (Fraser et al., 2010; Gaillard et al., 2014).

Maternal overnutrition during a critical period (intrauterine milieu and early lactation) may affect growth, metabolic‐relevant pathways, and tissue sensitivity to hormones and metabolites such as glucose, lipids, and amino acids in the fetus. The bioactive lipids induce inflammation independent of insulin sensitivity. The liver is a major organ responsible for metabolic reprogramming, particularly related to overnutrition. Previous studies have shown an important association between hepatic steatosis, fibrogenesis, and inflammation in maternal overnutrition (Oben et al., 2010; Wankhade et al., 2017). Adult male offspring of dams exposed to a high‐fat diet during pregnancy and lactation showed larger fat depots and increased serum levels of insulin, TNFα, and IL‐1β (Ashino et al., 2012). The liver triglyceride content in the offspring 1 week after weaning was increased and remained elevated into adulthood (Ashino et al., 2012). In addition, offspring of obese dams developed histological and biochemical changes compatible with hepatic steatosis with liver injury and fibrogenesis (Oben et al., 2010). However, the molecular mechanisms underlying the metabolic programming of offspring with either short‐ or long‐term consequences are poorly understood. Hence, the aim of this study was to investigate whether maternal intake of high‐fat diet (HFD) during the critical period of gestation and lactation affects metabolic health of offspring mice at weaning and in early adulthood.

## 2 MATERIALS AND METHODS

### 2.1 Animals

**FIGURE 1:**
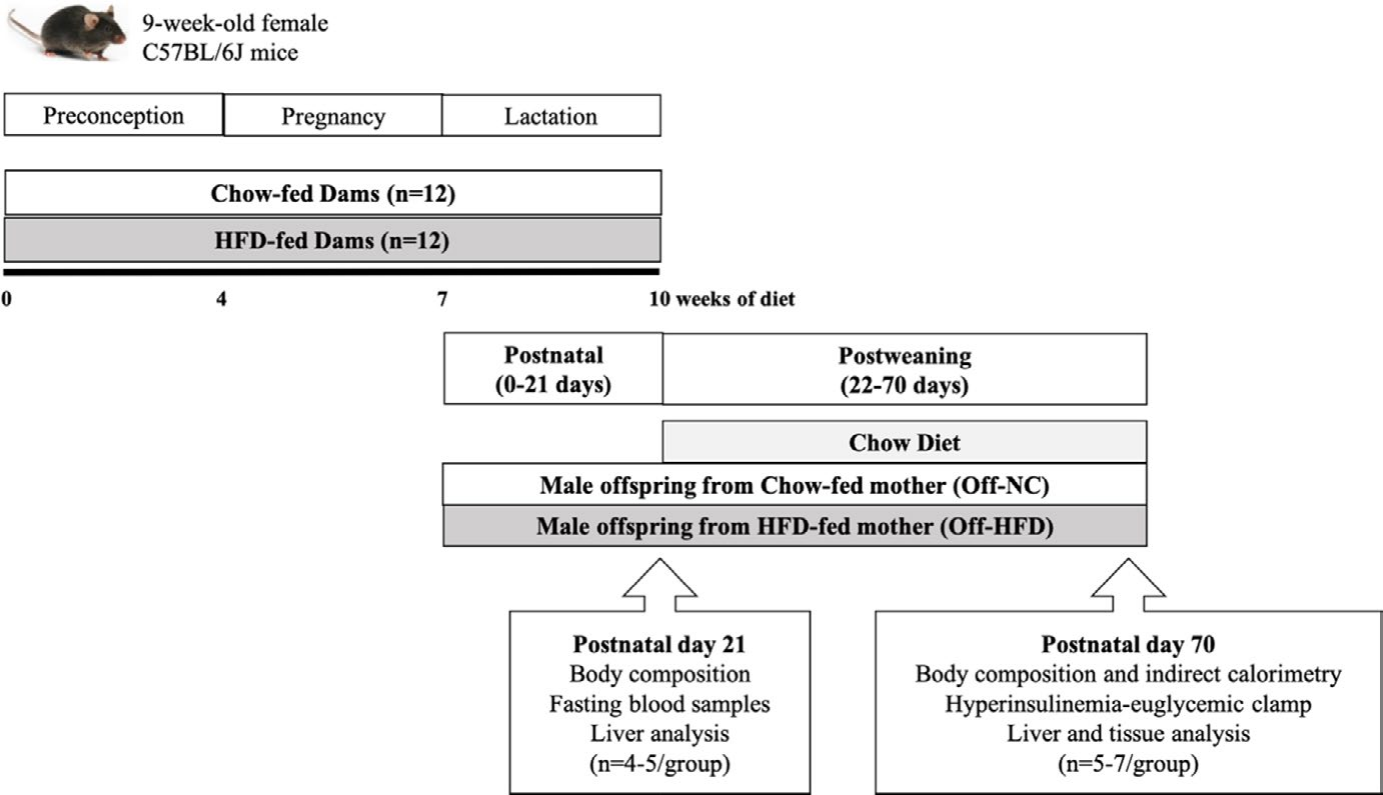
Study design and timeline of experimental groups. The control group (chow‐fed dams) was fed a standard chow diet (17% fat), while the HFD group (HFD‐fed dams) was fed a HFD (~60% fat by calories) for 4 weeks prior to mating and throughout pregnancy and lactation. Male offspring mice were housed with their mother until weaning. On postnatal day 21, offspring mice from HFD‐fed dams (Off‐HFD) and NC‐fed dams (Off‐NC) were weaned on a normal chow diet and remained on chow diet for the duration of the study. A subgroup of male offspring mice (n = 4~5/group) was used for fasting blood samples and liver analysis. At postnatal day 70, we performed the metabolic and molecular studies including the 2‐h hyperinsulinemic–euglycemic clamp and using tissues obtained at the end of the clamp from both groups of mice (n = 5~7/group)

Female C57BL/6 J mice at 9 weeks of age were purchased from The Jackson Laboratory (Bar Harbor, ME, USA) and maintained in a light‐ and temperature‐controlled facility (25°C, 12‐h light/dark cycle). The mice had free access to drinking water and were randomly assigned to either a high‐fat diet (HFD; 55% kcal from fat, 24% kcal from carbohydrate, and 21% kcal from protein; TD93075, Harlan Teklad) or standard chow diet (NC; 17% kcal from fat, 58% kcal from carbohydrate, and 25% kcal from protein; Prolab Isopro RMH3000 5P75; Labdiet) for 4 weeks before mating and during pregnancy and lactation (n = 12/group) (Figure 1).

Female mice were mated with age‐matched male chow‐fed C57BL/6 J mice. Body weights and body composition were measured weekly throughout gestation and lactation. According to sex differences in metabolic homeostasis and the potential effects of female sex hormones on energy homeostasis (Mauvais‐Jarvis et al., 2017), male offspring mice were used to determine the metabolic outcomes of maternal HFD exposure. Litter sizes were standardized to 5~7 pups at 72 h after delivery. Before the standardization of litters, all pups were individually weighed. Each litter contained an equal number of male pups of similar birth weight.

At postnatal day 21, male offspring mice from either HFD‐fed dams (Off‐HFD) or NC‐fed dams (Off‐NC) were weaned onto a normal chow diet, and metabolic and molecular experiments were performed 7 weeks later. A subgroup of male offspring mice (n = 4~5/group) was sacrificed after a 4‐h fast for blood chemistry and liver analysis. All of the experimental protocols were approved by the Institutional Animal Care and Use Committee of the University of Massachusetts Medical School.

### 2.2 Body composition and in vivo assessment of energy balance

Whole body fat and lean mass were noninvasively measured in mice using ^1^H‐magnetic resonance spectroscopy (MRS) (Echo‐MRI; Echo Medical System). Offspring body composition was monitored every 2 weeks. At postnatal day 60, indirectly calorimetry was noninvasively performed to assess energy balance (i.e., food/water intake, energy expenditure, respiratory quotient, and physical activity) for 3 days using metabolic cages (TSE Systems, Inc.).

### 2.3 Hyperinsulinemic–euglycemic clamp

At postnatal day 70, a survival surgery was performed in anesthetized mice to establish an indwelling catheter in the right internal jugular vein. After 4~5 days of recovery from surgery, a 2‐h hyperinsulinemic–euglycemic clamp was performed in awake mice to measure insulin sensitivity and glucose metabolism in individual organs (n = 5~7/group) (Kim, 2009). Following overnight fast, the clamp experiment began with a primed and continuous infusion of human insulin (150 mU/kg body weight bolus followed by 2.5 mU/kg per min) and a variable infusion of 20% glucose to maintain euglycemia in mice. Whole body glucose turnover was assessed with a continuous infusion of [3‐^3^H]glucose, and 2‐[^14^C]deoxyglucose was administered as a bolus (10 uCi) at 75 min of clamp experiments to measure insulin‐stimulated glucose uptake in individual organs. At the end of the clamp experiments, mice were euthanized, and tissues (liver, skeletal muscle [gastrocnemius], epidydimal white adipose tissue, and intrascapular brown adipose tissue) were snap‐frozen in liquid nitrogen and stored at −80°C for biochemical and molecular analysis (Kim, 2009).

### 2.4 Molecular analysis of hepatic gene expression

RNA from homogenized liver tissue was extracted using TRIzol reagent (Thermo Fisher Scientific) according to the manufacturer's protocols. cDNA was synthesized with the Omniscript kit (Qiagen). Quantitative real‐time polymerase chain reaction (RT‐qPCR) was performed using standard techniques for genes related to inflammation (IL‐6, MCP‐1, IL‐1β, TNFα), macrophage markers (CD68, F4/80), glucose transport and oxidation (GLUT4, IRS‐1, PDK4), gluconeogenesis (PEPCK, G6Pase), fatty acid transport and oxidation (CD36, FATP, PPARα), mitochondrial β‐oxidation (CPT1a), and de novo lipogenesis (FAS, SREBP1c, SCD1). Relative gene expression was calculated by using Bio‐Rad CFX manager software normalized to the hypoxanthine‐guanine phosphoribosyl transferase (HPRT) housekeeping gene.

### 2.5 Statistical analysis

Data are presented as means ± SE, and statistical differences between two groups were considered significant at *p* < 0.05 using student *t*‐test.

## 3 RESULTS

### 3.1 Effects of HFD on maternal metabolic profiles during pregnancy and lactation

After 4 weeks of HFD intervention, HFD‐fed dams had increased adiposity with significantly elevated whole body fat mass and percent weight gain compared to NC‐fed dams (Table 1). HFD‐fed dams showed type 2 diabetes phenotypes with significantly elevated fasting blood glucose levels (153.1 ± 5.7 vs. 111.8 ± 4.9 mg/dl in NC‐fed dams; *p* < 0.001). During the gestational period, HFD‐fed dams showed significantly lower percent weight gain and yielded smaller litter sizes compared with NC‐fed dams (Table 1). At the end of lactation (after 10 weeks of HFD exposure in the dams), total and percent weight gain were similar between the two groups of dams (2.9 ± 0.7 vs. 2.7 ± 0.8 g in NC‐fed dams, *p* = 0.86; percent weight gain, 10.2 ± 2.7 vs. 10.0 ± 3.5% in NC‐fed dams, *p* = 0.97). In contrast, HFD‐fed dams showed significant increases in whole body fat mass and fasting blood glucose levels compared to NC‐fed dams (Table 1).

**TABLE 1:** Maternal metabolic profiles at baseline (before HFD), after 4 weeks of HFD, during pregnancy (gestation), and at the end of lactation

	Chow‐fed dams (n = 12)	HFD‐fed dams (n = 12)	P value
Baseline			
Body weight (g)	21.8 ± 0.7	21.7 ± 0.7	0.91
Whole body fat mass (g)	2.8 ± 0.3	2.4 ± 0.2	0.22
After 4 weeks of HFD			
Body weight (g)	23.2 ± 0.6	26.6 ± 1.2	0.022
Whole body fat mass (g)	2.4 ± 0.4	5.7 ± 0.8	0.001
Fasting blood glucose (mg/dl)	111.8 ± 4.9	153.1 ± 5.7	<0.001
Gestation			
Gestational weight gain (g)	17.0 ± 0.5	15.9 ± 0.7	0.257
Percent weight gain (%)	72.4 ± 2.8	61.6 ± 4.2	0.047
Litter size (n)	7.3 ± 1	3.8 ± 0.8	0.018
End of Lactation			
Body weight (g)	31.4 ± 0.7	32.2 ± 1.4	0.62
Whole body fat mass (g)	3.4 ± 0.3	6.2 ± 0.7	0.002
Fasting blood glucose (mg/dl)	107.1 ± 5.0	139.8 ± 7.1	0.002

The results are expressed as means ± SEM. Data were analyzed by Student's *t*‐test.

### 3.2 Effects of maternal HFD in utero and during lactation on male offspring growth

**FIGURE 2:**
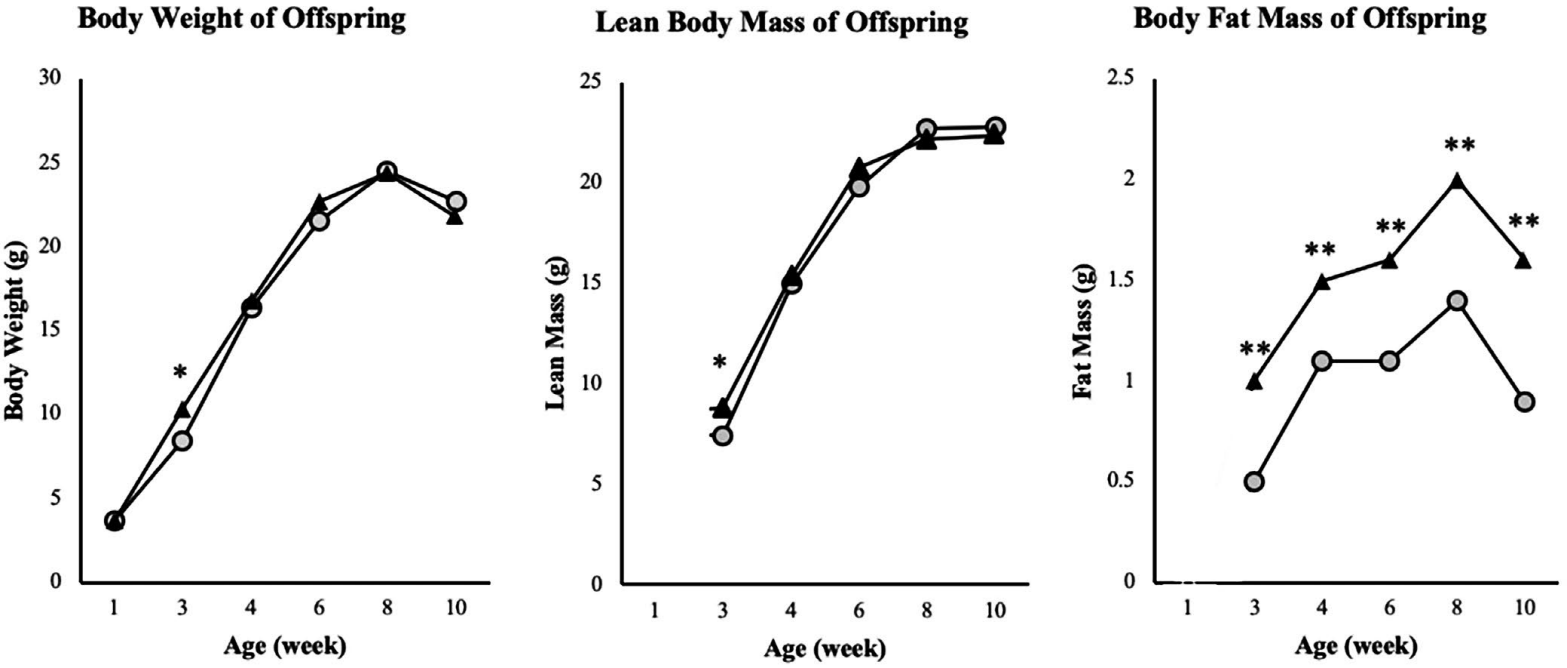
Changes in body composition of male offspring mice. The longitudinal changes in body weight and whole body lean and fat mass (measured using ^1^H‐MRS) in male offspring mice fed chow diet through 10 weeks of age. *p‐value <0.05, **p‐value <0.01

Following birth, we monitored the weekly weight gain of male offspring mice from postnatal day 7 to day 70. Male offspring mice from HFD‐fed dams (Off‐HFD; n = 14) and male offspring mice from NC‐fed dams (Off‐NC; n = 31) showed similar body weights at postnatal day 7 (Figure 2). By postnatal day 21, Off‐HFD mice gained more weight (6.1 ± 0.4 vs. 5.0 ± 0.1 g; *p* = 0.003), and this was due to significant increases in whole body fat mass (1.0 ± 0.1 vs. 0.6 ± 0.03 g; *p* < 0.0001) and lean mass (8.8 ± 0.5 vs. 7.4 ± 0.3 g; *p* = 0.015) in Off‐HFD mice compared to Off‐NC mice (Figure 2). Additionally, fasting levels of glucose, insulin, and cholesterol were markedly elevated in Off‐HFD mice compared to Off‐NC mice (Table 2). Some of these effects were transient as the whole body lean mass between Off‐HFD mice and Off‐NC mice became comparable afterwards and through 10 weeks of age. In contrast, whole body fat mass remained higher in Off‐HFD mice compared to Off‐NC mice through 10 weeks of age. While there were no differences in circulating and liver triglyceride levels between the two groups (Table 2), the metabolic changes in Off‐HFD mice reflect the onset of type 2 diabetes phenotypes.

**TABLE 2:** Metabolic parameters of male offspring mice at weaning

	Off‐NC (n = 5)	Off‐HFD (n = 5)	P value
Fasting blood glucose (mg/dl)	139.6 ± 6.4 (n = 15)	184.0 ± 8.2 (n = 15)	<0.001
Insulin (ng/ml)	0.23 ± 0.1	1.05 ± 0.2	0.005
Cholesterol (mg/dl)	60.2 ± 3.6	80.0 ± 5.6	0.017
Triglyceride (mg/dl)	50.6 ± 5.4	48.3 ± 10.2	0.83
Liver triglyceride (μmol/g of liver tissue)	46.1 ± 2.5	40.3 ± 4.5	0.27

Note:

The results are expressed as means ± SEM. Data were analyzed by Student's *t*‐test.

Abbreviations: Off‐HFD, offspring mice from high‐fat diet‐fed dams; Off‐NC, offspring mice from chow‐fed dams.

### 3.3 Inflammatory and metabolic gene expression in liver of male offspring mice at postnatal day 21

**FIGURE 3:**
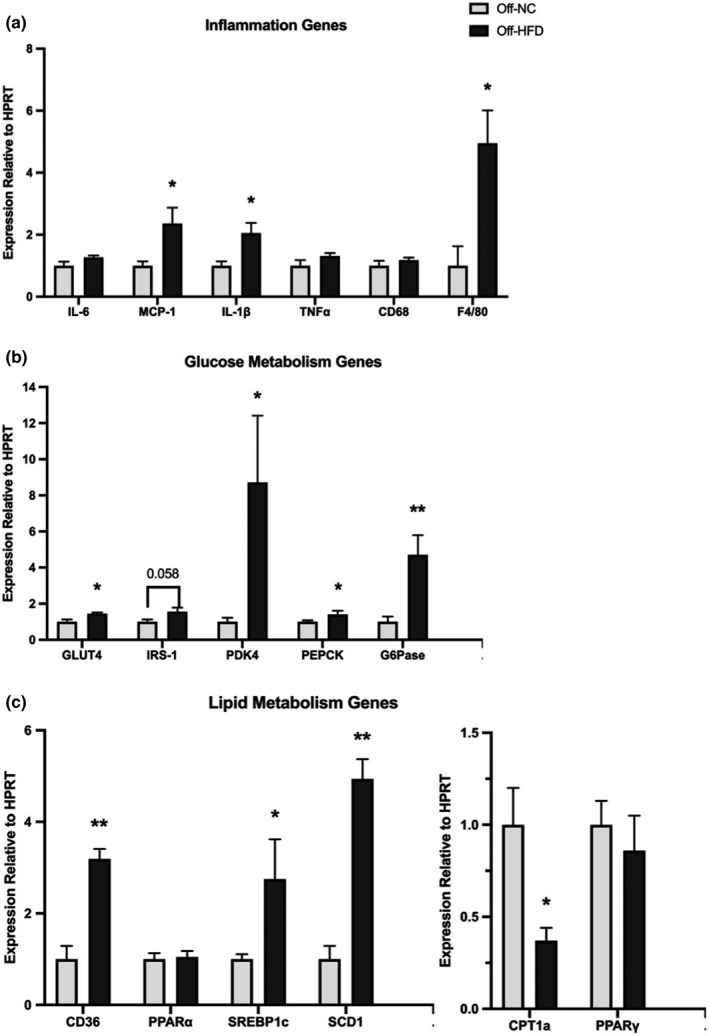
Liver expression of inflammatory and metabolic genes in male offspring mice at postnatal day 21. Liver expression of genes associated with (a) Inflammation and macrophages, (b) glucose metabolism, and (c) lipid metabolism in male offspring mice from HFD‐fed dams (Off‐HFD) compared to male offspring mice from normal chow‐fed dams (Off‐NC) at postnatal day 21 (n = 4~5/group). Data were analyzed by Student's *t*‐test and expressed as means ± SEM. Abbreviations: CD36, Cluster of Differentiation 36; CD68, Cluster of Differentiation 68; CPT1a, Carnitine Palmitoyltransferase 1a; FAS, Fatty Acid Synthase; FATP1, Fatty Acid Transport Protein 1; GLUT4, Glucose Transporter type 4; G6Pase, Glucose‐6‐phosphatase; IL‐1β, Interleukin 1 beta; IL‐6, Interleukin 6; IRS‐1, Insulin Receptor Substrate 1; MCP‐1, Monocyte Chemoattractant Protein 1; PDK4, Pyruvate Dehydrogenase Kinase 4; PEPCK, Phosphoenolpyruvate Carboxykinase; PPARα, Peroxisome Proliferator‐activated Receptor alpha; PPARγ, Peroxisome Proliferator‐activated Receptor gamma; SCD1, Stearoyl CoA Desaturase 1; SREBP1c, Sterol Response Element‐binding Protein 1c; TNFα, Tumor Necrosis Factor alpha. *p‐value <0.05, **p‐value <0.01

To determine the early effects of maternal HFD exposure on offspring liver, we collected liver samples from a cohort Off‐HFD and Off‐NC mice at weaning (postnatal day 21). We were surprised to find dramatic changes in liver expression of genes associated with inflammation and metabolism in young Off‐HFD mice. The liver mRNA levels of chemokine (*MCP‐1*), cytokine (*IL‐1β*), and macrophages (*F4/80*) were significantly increased by two‐ to fivefold in Off‐HFD mice compared to Off‐NC mice (Figure 3a). Compared to Off‐NC mice, hepatic expression of gluconeogenic genes, namely *G6Pase* and *PEPCK*, was also significantly elevated by five and twofold, respectively (*p* < 0.05), in Off‐HFD mice compared to Off‐NC mice, and this was consistent with fasting hyperglycemia in these mice (Figure 3b). Hepatic expression of genes associated with glucose metabolism (*GLUT4* and *PDK4*), insulin signaling (*IRS*‐*1*), and lipid metabolism (*CD36*, *SREBP1c*, and *SCD1*) were all significantly elevated in Off‐HFD mice (Figure 3b,c). In contrast, liver mRNA levels of *CPT1a* were markedly reduced by ~60% in Off‐HFD mice (*p* = 0.029), and those of *PPARα* and *PPARγ* were not affected in Off‐HFD mice (Figure 3c). Taken together, these data indicate that maternal exposure of HFD during pregnancy and lactation promotes hepatic inflammation and alterations in hepatic glucose and lipid metabolism in male offspring mice.

### 3.4 Long‐term effects of maternal HFD exposure on the metabolic health of male offspring mice

To determine the long‐term effects of maternal exposure of HFD during a critical period before, during, and after pregnancy, Off‐HFD and Off‐NC mice were weaned onto a normal chow diet and remained on chow diet through 10 weeks of age. From weaning through postnatal day 70, Off‐HFD and Off‐NC mice showed similar body weights (Figure 2). However, a careful analysis of body composition found that Off‐HFD mice had modestly but significantly higher whole body fat mass (1.6 ± 0.2 vs. 0.9 ± 0.1 g in Off‐NC mice; *p* < 0.005) compared with Off‐NC mice from weaning through postnatal day 70 (Figure 2). At postnatal day 60, we performed a 3‐day analysis of energy balance using metabolic cages and found no significant differences in daily food intake, physical activity, or energy expenditure between groups (Figure S1).

**FIGURE 4:**
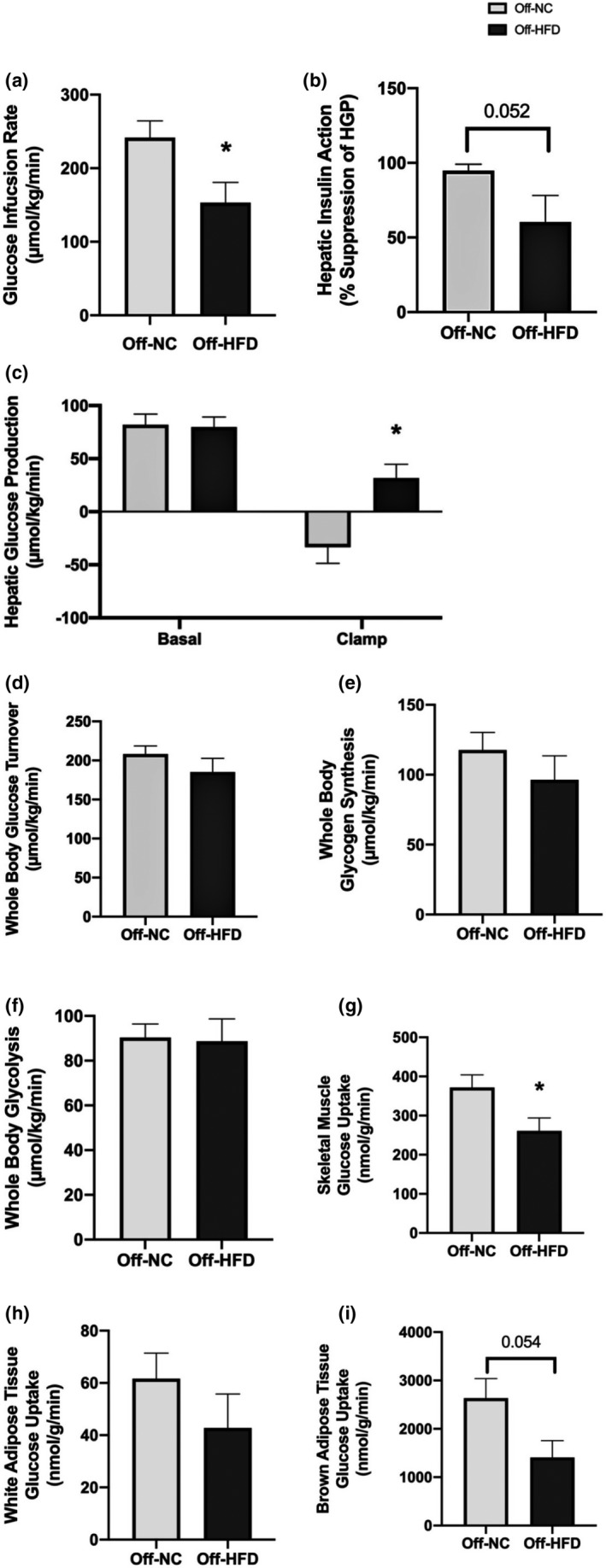
Insulin action and glucose metabolism in male offspring mice at postnatal day 70. Insulin action and glucose metabolism during a hyperinsulinemic–euglycemic clamp in Off‐NC and Off‐HFD mice on chow diet at postnatal day 70. (a) Glucose infusion rate, (b) hepatic insulin action, (c) hepatic glucose production, (d) whole body glucose turnover, (e) whole body glycogen synthesis, (f) whole body glycolysis, (g) skeletal muscle glucose uptake, (h) white adipose tissue glucose uptake, and (i) brown adipose tissue uptake. Data were analyzed by Student's *t*‐test. Data are expressed as the mean ± SEM

Next, we performed a 2‐h hyperinsulinemic–euglycemic clamp to measure insulin sensitivity and glucose metabolism in young adult male offspring mice (postnatal day 70; n = 5~7/group). Despite similar body weights, Off‐HFD mice developed insulin resistance as indicated by a 36% decrease in steady‐state glucose infusion rates to maintain euglycemia during clamps compared with Off‐NC mice (154 ± 27 vs. 242 ± 23 µmol/kg/min; *p* = 0.032) (Figure 4a). This was partly due to insulin resistance in the liver as hepatic insulin action, represented as insulin‐mediated percent suppression of basal hepatic glucose production (HGP), showed a strong tendency to be reduced in Off‐HFD mice compared to Off‐NC mice (*p* = 0.052) (Figure 4b). Basal HGP rates did not differ between groups, but clamp HGP rates were significantly elevated in Off‐HFD mice compared to Off‐NC mice as insulin failed to suppress HGP in Off‐HFD mice (Figure 4c). Whole body glucose turnover, glycogen synthesis, and glycolysis tended to be reduced in Off‐HFD mice, but these differences did not reach statistical significance (Figure 4d–f). In contrast, insulin‐stimulated glucose uptake in skeletal muscle (gastrocnemius) was significantly reduced by 30% in Off‐HFD mice, and insulin‐stimulated glucose uptake in brown adipose tissue (intrascapular) and white adipose tissue (epidydimal) tended to be reduced by 30–50% in Off‐HFD mice compared with Off‐NC mice (Figure 4g–i).

### 3.5 Long‐term effects of maternal HFD exposure on liver inflammation and metabolism in male offspring mice

**FIGURE 5:**
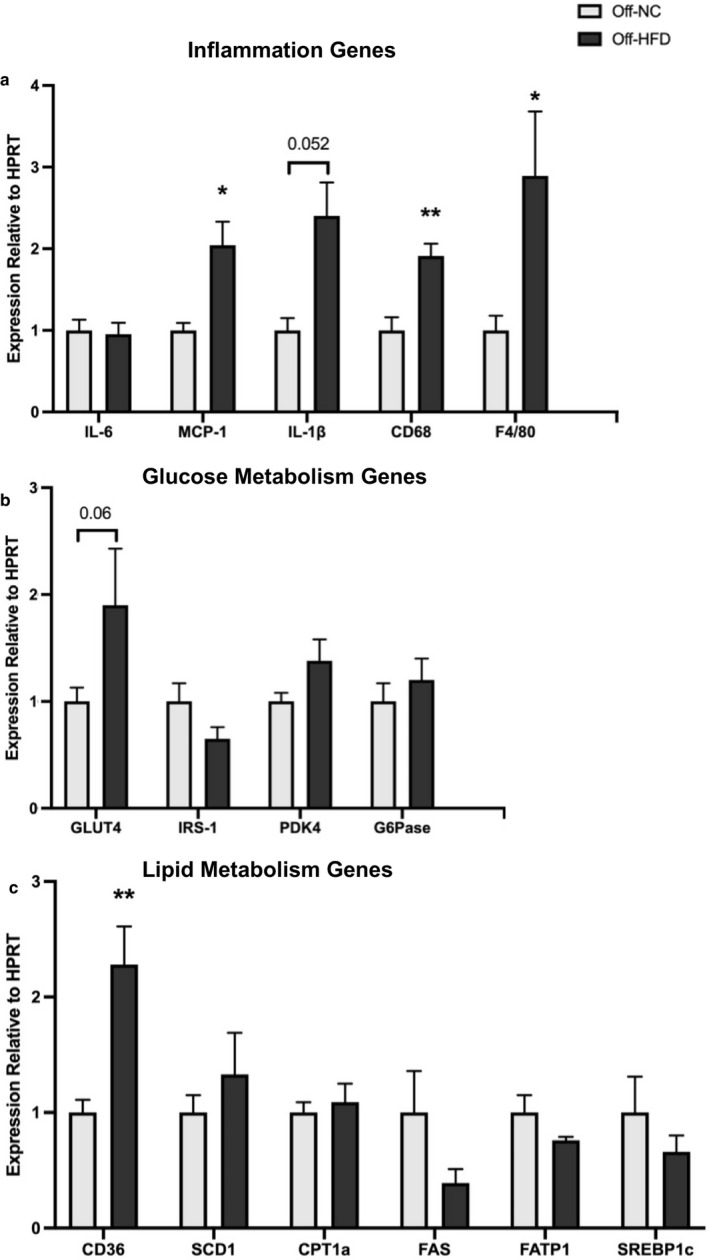
Liver expression of inflammatory and metabolic genes in male offspring mice at postnatal day 70. Hepatic gene expression of (a) inflammatory and macrophage markers, (b) glucose metabolism, and (c) lipid metabolism were determined by RT‐qPCR (n = 5~7/group). Data were analyzed by Student's *t*‐test. Data are expressed as the mean ± SEM. *p‐value <0.05, **p‐value <0.01

We performed RT‐qPCR analysis in liver samples obtained from male offspring mice at postnatal day 70 to assess the inflammatory and metabolic changes in gene expression. Liver mRNA levels of chemokine (MCP‐1) and macrophage markers (CD68 and F4/80) were significantly increased in Off‐HFD mice compared to Off‐NC mice, suggesting hepatic inflammation in these mice (Figure 5a). Liver expression of inflammatory cytokine, IL‐1β, also tended to be elevated in Off‐HFD mice (*p* = 0.052). Furthermore, liver mRNA levels of genes associated with glucose metabolism (*GLUT4*, *PDK4*, and *G6Pase*) were increased in Off‐HFD mice compared with Off‐NC mice, and hepatic *IRS*‐*1* expression tended to be higher in Off‐HFD mice (Figure 5b). However, these findings did not reach statistical significance. There were also major changes in hepatic lipid metabolic genes as liver mRNA levels of *CD36* and *SCD1* were markedly increased in Off‐HFD mice compared with Off‐NC mice (Figure 5c).

## 4 DISCUSSION

The rising prevalence of overweight and obesity has been considered a global pandemic in all age groups, including children. Recent studies found that childhood obesity and type 2 diabetes have origins in utero (Lawlor et al., 2006; Rogers, 2005). Understanding the pathological consequences in children exposed to nutrient insult during a critical period is important for developing interventions to prevent obesity and metabolic diseases later in life. Our data suggest that maternal exposure to a HFD caused an early increase in adiposity and fasting glucose and insulin levels at weaning in male offspring mice. These findings are consistent with previous reports on the important association between HFD exposure during the intrauterine and postnatal periods and increased birth weight, weight gain, fat mass, and prevalence of type 2 diabetes phenotypes in offspring (Ainge et al., 2011; Ribaroff et al., 2017). However, Kruse M et al. showed that offspring mice from HFD‐fed dams had a slightly smaller body weight up to the age of 6 weeks, but no significant differences were observed in adiposity and fasting blood glucose and insulin levels at 26 weeks of age (Kruse et al., 2013). The discordant results may be due to a shorter duration of HFD exposure when compared to our current study.

Previous studies have reported that an increased dietary fat intake in an adult mouse model was associated with increased fat deposition in the liver, leading to nonalcoholic fatty liver disease and progression to more advanced hepatic fibrosis and cirrhosis (Fraulob et al., 2010; Hebbard & George, 2011; Zou et al., 2006). Similar to these results, Patridge CG et al. found that a chronic feeding of HFD (42% kcal from fat) from weaning to 20 weeks of age significantly upregulated lipid and carbohydrate metabolic genes as well as inflammatory genes in liver (Partridge et al., 2014). In the present study, male offspring mice from HFD‐fed dams showed increased inflammatory state of the liver with upregulation of proinflammatory genes and macrophage markers. Additionally, fasting hyperglycemia and hyperinsulinemia in 21‐day‐old Off‐HFD mice can be attributed to alterations in liver expression of genes associated with glucose transport (GLUT4), insulin signaling (IRS‐1), oxidative glucose metabolism (PDK4), and gluconeogenesis (PEPCK, G6Pase). Under diabetic conditions, the PDK4 genes are upregulated, resulting in increased gluconeogenesis in the liver and decreased glucose utilization in peripheral tissues. A recent study from Tao et al. supported this finding that deletion of the PDK4 gene reduced glycemia and improved glucose tolerance compared with knockout of the PDK2 gene on the IRS1/2 knockout genetic background (Tao et al., 2013). A meta‐analysis of lipid profiles in male offspring mice of HFD‐fed dams revealed a significant increase in triglyceride and cholesterol levels (Ribaroff et al., 2017). Consistent with this, we observed that maternal overnutrition during pregnancy and lactation affected hepatic lipid metabolism. The abnormally increased rate of de novo fatty acid synthase (SCD1, SREBP1c) contributes to a significant increase in plasma cholesterol but not triglyceride levels. In contrast, mitochondrial β‐oxidation was decreased in Off‐HFD mice, as evidenced by a marked reduction in CPT1a expression, which is a rate‐limiting enzyme of mitochondrial β‐oxidation. Our results are consistent with a prior study from Benatti RO et al. showing a larger adipose tissue mass, more insulin resistance, and higher cholesterol and triglyceride levels in Off‐HFD mice compared to controls (Benatti et al., 2014). They also demonstrated that hepatic mRNA expression of SCD1 was significantly increased, whereas CPT1a gene expression was decreased in weaned Off‐HFD mice (on day 28).

Numerous reports have focused on understanding the metabolic consequences of maternal HFD exposure in early life and late adulthood, in terms of body weight, body composition, and type 2 diabetes phenotypes. Our study demonstrated that the early increase in body weight of Off‐HFD mice at the time of weaning was quickly normalized within 2 weeks after weaning onto a standard chow diet, which could be due to cessation of a high‐fat intake via lactation. Based on weight monitoring from weaning to 10 weeks of age, male offspring mice from HFD‐fed dams showed a lasting change in the body's fat proportions with higher fat mass compared to offspring mice from NC‐fed dams. However, it is important to point out that this small difference in whole body fat mass (less than 1 g) is unlikely responsible for obesity‐mediated changes in glucose metabolism, typically observed in HFD‐fed mice with 20% or more of percent body fat. Moreover, we showed that many of the effects of maternal HFD exposure in inducing obesity and type 2 diabetes phenotypes were normalized after switching to a standard chow diet that included a reduction in total body weight and fasting plasma glucose and similar energy expenditure, physical activity, and daily dietary intakes in early adulthood. These findings are consistent with previous data from Chang et al. that showed no significant differences in body weight, fasting insulin levels, and glucose tolerance in 12‐week‐old male offspring mice from mothers that were exposed to HFD during pregnancy and gestational periods (Chang et al., 2019). However, the study of Samuelsson et al. (Samuelsson et al., 2008) reported hyperphagia and physical inactivity in 3‐week‐old offspring mice from obese dams.

Offspring mice from HFD‐fed dams showed no significant differences in fasting blood glucose levels and glucose response during an oral glucose tolerance test at 10 ~ 12 weeks of age (Buckley et al., 2005 Apr 1; Tamashiro et al., 2009 May 1) as well as whole body insulin sensitivity (Buckley et al., 2005 Apr 1). However, it is important to point out that although Off‐HFD mice did not show fasting hyperglycemia or hyperinsulinemia, and insulin sensitivity was altered in individual organs. Our data from the hyperinsulinemic–euglycemic clamp, a gold‐standard method of measuring insulin sensitivity, showed that Off‐HFD mice at 10 weeks of age developed systemic insulin resistance that was due to significant reductions in hepatic insulin action and insulin‐stimulated glucose uptake in skeletal muscle and brown adipose tissue.

Maternal overnutrition in animal models has been associated with impaired glucose tolerance from a reduction in ß‐cell number, volume, and insulin secretory capacity (Cerf et al., 2005; Taylor et al., 2005). Additionally, our study showed important findings that liver inflammation as well as impaired hepatic glucose and lipid metabolism may contribute to type 2 diabetes phenotypes in the early life of male offspring mice. This study also highlighted the consequences of perinatal overnutrition in young adult offspring. The Off‐HFD mice showed a higher whole body fat mass ratio that lasted through postnatal day 70. The dramatic normalization of glucose metabolism and gluconeogenic gene expression levels at postnatal day 21 resulted from the cessation of HFD exposure via lactation in Off‐HFD mice. However, a pronounced upregulation of macrophage markers, proinflammatory cytokines, and fatty acid transport genes was observed in Off‐HFD mice, indicating persistent hepatic inflammation in young adult offspring mice. Another important finding from our study is that young adult Off‐HFD mice developed insulin resistance with failure to suppress hepatic glucose production as well as reduced insulin‐mediated glucose uptake in skeletal muscle and brown adipose tissue.

Some limitations should be noted. First, we used a standard chow diet without customization as a control diet that has different concentrations of micronutrients and vitamins, and it is possible that the effects on the offspring could be due to differences in the nutrient concentrations of the diets. Second, the maternal animals in the HFD group had hyperglycemia, and some of the metabolic effects in the offspring mice may be attributed to the lasting effects of early exposure to maternal hyperglycemia. There are a number of confounding variables in the experiments that could affect offspring outcomes, such as the small litter size at birth, resulting in less competition during lactation. Both of these factors have been shown to result in increased adiposity and impaired glucose tolerance in offspring independent of maternal diet.

In summary, despite being normal weight, there are hidden, lasting effects of maternal exposure to a HFD during pregnancy and lactation on liver inflammation and whole body glucose metabolism in male offspring mice. These effects may be exacerbated by the “second” hit of HFD exposure in later life that may contribute to the current obesity pandemic. Our findings strongly support the growing need to understand the mechanistic link between maternal nutrient intake and metabolic health of offspring in later life as the potential mechanisms may involve epigenetic modifications, the gut microbiome, and metabolomics, and further highlight the importance of implementing an appropriate dietary intervention during the critical period of gestation and lactation.

## CONFLICT OF INTEREST

The authors have no conflicts to disclose.

## AUTHORS’ CONTRIBUTION

Conception: Saengnipanthkul S and Kim JK. Data collection: Saengnipanthkul S, Noh HL, Friedline RH, Suk S, Choi S, Acosta NK, Tran DA, Hu X, Inashima K and Kim AM. Data analysis and interpretation: Saengnipanthkul S, Noh HL, Friedline RH, Hu X and Kim JK. Drafting the article: Saengnipanthkul K. Critical revision of the article: Kim JK and Lee KW. Final approval: Kim JK.

## ETHICAL STANDARDS

The authors assert that all procedures contributing to this work comply with the ethical standards of the Institutional Animal Care and Use Committee of the University of Massachusetts Medical School.

## Supporting information



Fig S1

## References

[phy214811-bib-0001] Ainge, H. , Thompson, C. , Ozanne, S. E. , & Rooney, K. B. (2011). A systematic review on animal models of maternal high fat feeding and offspring glycaemic control. International Journal of Obesity, 35(3), 325–335. 20680016 10.1038/ijo.2010.149

[phy214811-bib-0002] Ashino, N. G. , Saito, K. N. , Souza, F. D. , Nakutz, F. S. , Roman, E. A. , Velloso, L. A. , Torsoni, A. S. , & Torsoni, M. A. (2012). Maternal high‐fat feeding through pregnancy and lactation predisposes mouse offspring to molecular insulin resistance and fatty liver. The Journal of Nutritional Biochemistry, 23(4), 341–348. 21543214 10.1016/j.jnutbio.2010.12.011

[phy214811-bib-0003] Barker, D. J. P. (2007). The origins of the developmental origins theory. Journal of Internal Medicine, 261(5), 412–417. 17444880 10.1111/j.1365-2796.2007.01809.x

[phy214811-bib-0004] Benatti, R. O. , Melo, A. M. , Borges, F. O. , Ignacio‐Souza, L. M. , Simino, L. A. P. , Milanski, M. (2014). Maternal high‐fat diet consumption modulates hepatic lipid metabolism and microRNA‐122 (miR‐122) and microRNA‐370 (miR‐370) expression in offspring. British Journal of Nutrition, 111(12), 2112–2122. 10.1017/S0007114514000579 24666709

[phy214811-bib-0005] Buckley, A. J. , Keserü, B. , Briody, J. , Thompson, M. , Ozanne, S. E. , & Thompson, C. H. (2005). Altered body composition and metabolism in the male offspring of high fat–fed rats. Metabolism, 54(4), 500–507. 15798958 10.1016/j.metabol.2004.11.003

[phy214811-bib-0006] Cerf, M. E. , Williams, K. , Nkomo, X. I. , Muller, C. J. , Du Toit, D. F. , Louw, J. , & Wolfe‐Coote, S. A. (2005). Islet cell response in the neonatal rat after exposure to a high‐fat diet during pregnancy. American Journal of Physiology‐Regulatory, Integrative and Comparative Physiology, 288(5), R1122–R1128. 10.1152/ajpregu.00335.2004 15705804

[phy214811-bib-0007] Chang, E. , Hafner, H. , Varghese, M. , Griffin, C. , Clemente, J. , Islam, M. , Carlson, Z. , Zhu, A. , Hak, L. , Abrishami, S. , Gregg, B. , & Singer, K. (2019). Programming effects of maternal and gestational obesity on offspring metabolism and metabolic inflammation. Scientific Reports, 9(1), 16027. 31690792 10.1038/s41598-019-52583-x PMC6831633

[phy214811-bib-0008] Fraser, A. , Tilling, K. , Macdonald‐Wallis, C. , Sattar, N. , Brion, M.‐J. , Benfield, L. , Ness, A. , Deanfield, J. , Hingorani, A. , Nelson, S. M. , Smith, G. D. , & Lawlor, D. A. (2010). Association of maternal weight gain in pregnancy with offspring obesity and metabolic and vascular traits in childhood. Circulation, 121(23), 2557–2564. 20516377 10.1161/CIRCULATIONAHA.109.906081 PMC3505019

[phy214811-bib-0009] Fraulob, J. C. , Ogg‐Diamantino, R. , Fernandes‐Santos, C. , Aguila, M. B. , & Mandarim‐de‐Lacerda, C. A. (2010). A mouse model of metabolic syndrome: Insulin resistance, fatty liver and non‐alcoholic fatty pancreas disease (NAFPD) in C57BL/6 mice fed a high fat diet. Journal of Clinical Biochemistry and Nutrition, 46(3), 212–223. 20490316 10.3164/jcbn.09-83 PMC2872226

[phy214811-bib-0010] Gaillard, R. , Steegers, E. A. P. , Duijts, L. , Felix, J. F. , Hofman, A. , Franco, O. H. , & Jaddoe, V. W. V. (2014). Childhood cardiometabolic outcomes of maternal obesity during pregnancy: The generation R study. Hypertension, 63(4), 683–691. 24379180 10.1161/HYPERTENSIONAHA.113.02671

[phy214811-bib-0011] Hebbard, L. , & George, J. (2011). Animal models of nonalcoholic fatty liver disease. Nature Reviews Gastroenterology & Hepatology, 8(1), 35–44. 21119613 10.1038/nrgastro.2010.191

[phy214811-bib-0012] Kim, J. K. (2009). Hyperinsulinemic‐euglycemic clamp to assess insulin sensitivity in vivo. Methods in Molecular Biology, 560, 221–238. 19504253 10.1007/978-1-59745-448-3_15

[phy214811-bib-0013] Kruse, M. , Seki, Y. , Vuguin, P. M. , Du, X. Q. , Fiallo, A. , Glenn, A. S. , Singer, S. , Breuhahn, K. , Katz, E. B. , & Charron, M. J. (2013). High‐fat intake during pregnancy and lactation exacerbates high‐fat diet‐induced complications in male offspring in mice. Endocrinology, 154(10), 3565–3576. 23861375 10.1210/en.2012-1877 PMC3776861

[phy214811-bib-0014] Lawlor, D. A. , Smith, G. D. , O’Callaghan, M. , Alati, R. , Mamun, A. A. , Williams, G. M. , & Najman, J. M. (2006). Epidemiologic evidence for the fetal overnutrition hypothesis: Findings from the Mater‐University Study of pregnancy and its outcomes. American Journal of Epidemiology, 165(4), 418–424. 17158475 10.1093/aje/kwk030

[phy214811-bib-0015] Mauvais‐Jarvis, F. , Arnold, A. P. , & Reue, K. (2017). A Guide for the design of pre‐clinical studies on sex differences in metabolism. Cell Metabolism, 25, 1216–1230. 28591630 10.1016/j.cmet.2017.04.033 PMC5516948

[phy214811-bib-0016] Oben, J. A. , Mouralidarane, A. , Samuelsson, A.‐M. , Matthews, P. J. , Morgan, M. L. , McKee, C. , Soeda, J. , Fernandez‐Twinn, D. S. , Martin‐Gronert, M. S. , Ozanne, S. E. , Sigala, B. , Novelli, M. , Poston, L. , & Taylor, P. D. (2010). Maternal obesity during pregnancy and lactation programs the development of offspring non‐alcoholic fatty liver disease in mice. Journal of Hepatology, 52(6), 913–920. 20413174 10.1016/j.jhep.2009.12.042

[phy214811-bib-0017] Partridge, C. G. , Fawcett, G. L. , Wang, B. , Semenkovich, C. F. , & Cheverud, J. M. (2014). The effect of dietary fat intake on hepatic gene expression in LG/J AND SM/J mice. BMC Genomics, 15(1), 99. 24499025 10.1186/1471-2164-15-99 PMC4028868

[phy214811-bib-0018] Ribaroff, G. A. , Wastnedge, E. , Drake, A. J. , Sharpe, R. M. , & Chambers, T. J. G. (2017). Animal models of maternal high fat diet exposure and effects on metabolism in offspring: a meta‐regression analysis. Obesity Reviews, 18, 673–686. 28371083 10.1111/obr.12524 PMC5434919

[phy214811-bib-0019] Rogers, I. (2005). Birth weight and obesity and fat distribution in later life. Birth Defects Research Part A: Clinical and Molecular Teratology, 73(7), 485–486. 15959890 10.1002/bdra.20168

[phy214811-bib-0020] Samuelsson, A.‐M. , Matthews, P. A. , Argenton, M. , Christie, M. R. , McConnell, J. M. , Jansen, E. H. J. M. , Piersma, A. H. , Ozanne, S. E. , Twinn, D. F. , Remacle, C. , Rowlerson, A. , Poston, L. , & Taylor, P. D. (2008). Diet‐induced obesity in female mice leads to offspring hyperphagia, adiposity, hypertension, and insulin resistance. Hypertension, 51(2), 383–392. 18086952 10.1161/HYPERTENSIONAHA.107.101477

[phy214811-bib-0021] Tamashiro, K. L. K. , Terrillion, C. E. , Hyun, J. , Koenig, J. I. , & Moran, T. H. (2009). Prenatal stress or high‐fat diet increases susceptibility to diet‐induced obesity in rat offspring. Diabetes 58(5), 1116–1125. 19188431 10.2337/db08-1129 PMC2671057

[phy214811-bib-0022] Tao, R. , Xiong, X. , Harris, R. A. , White, M. F. , & Dong, X. C. (2013). Genetic inactivation of pyruvate dehydrogenase kinases improves hepatic insulin resistance induced diabetes. PLoS One, 8(8), e71997. 23940800 10.1371/journal.pone.0071997 PMC3733847

[phy214811-bib-0023] Taylor, P. D. , McConnell, J. , Khan, I. Y. , Holemans, K. , Lawrence, K. M. , Asare‐Anane, H. , Persaud, S. J. , Jones, P. M. , Petrie, L. , Hanson, M. A. , & Poston, L. (2005). Impaired glucose homeostasis and mitochondrial abnormalities in offspring of rats fed a fat‐rich diet in pregnancy. American Journal of Physiology‐Regulatory, Integrative and Comparative Physiology, 288(1), R134–R139. 10.1152/ajpregu.00355.2004 15388492

[phy214811-bib-0024] Wankhade, U. D. , Zhong, Y. , Kang, P. , Alfaro, M. , Chintapalli, S. V. , Thakali, K. M. , & Shankar, K. . (2017). Enhanced offspring predisposition to steatohepatitis with maternal high‐fat diet is associated with epigenetic and microbiome alterations. PLoS One, 12(4), e0175675. 28414763 10.1371/journal.pone.0175675 PMC5393586

[phy214811-bib-0025] Zou, Y. , Li, J. , Lu, C. , Wang, J. , Ge, J. , Huang, Y. , Zhang, L. , & Wang, Y. (2006). High‐fat emulsion‐induced rat model of nonalcoholic steatohepatitis. Life Sciences, 79(11), 1100–1107. 16624332 10.1016/j.lfs.2006.03.021

