# Supplementary material for: Maternal exposure to high‐fat diet during pregnancy and lactation predisposes normal weight offspring mice to develop hepatic inflammation and insulin resistance
Source: Physiol Rep. 2021 Mar 26;9(6):e14811. doi: 10.14814/phy2.14811 (PMC7995551; doi:10.14814/phy2.14811)
Supplement: Supplementary file 1 — Fig S1 [file PHY2-9-e14811-s001.docx]

**Supplement Fig 1. Energy balance and food intake of male offspring mice at postnatal day 60.**

*
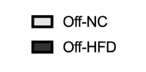
*

*a b c*


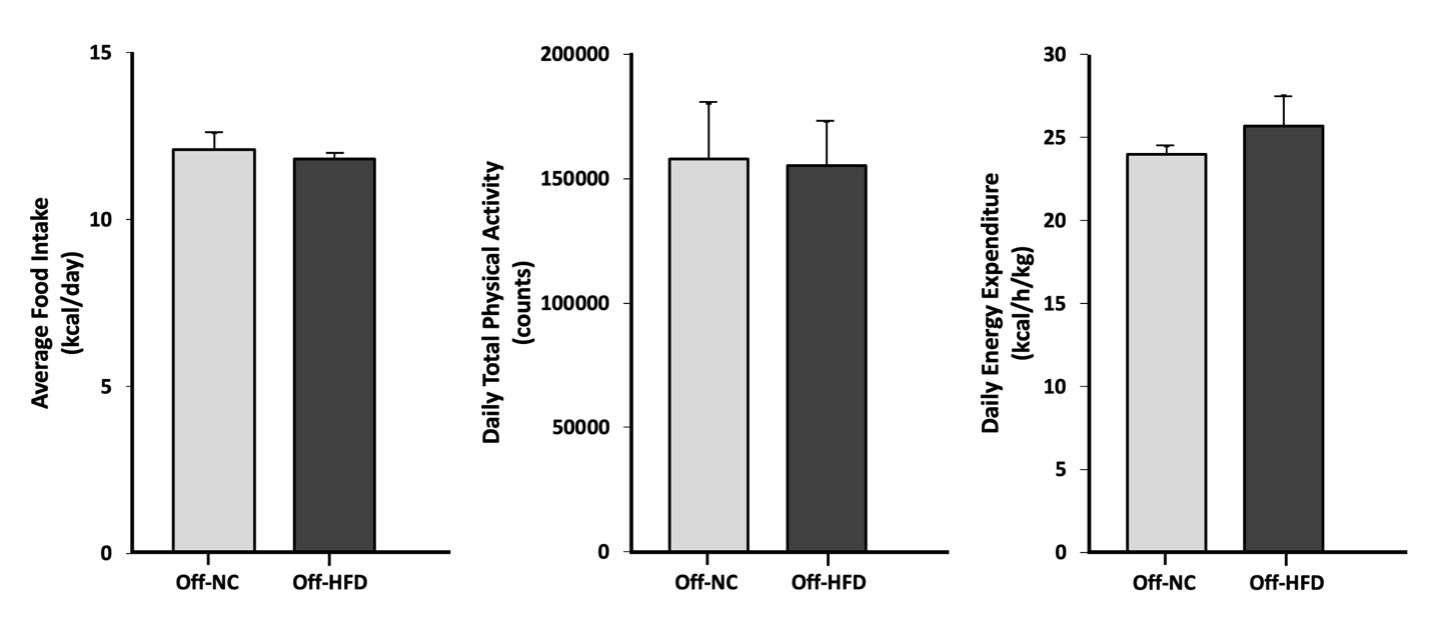


(A) Average food intake, (B) daily total physical activity, and (C) daily energy expenditure were assessed in male offspring mice at postnatal day 60 for 3 days using metabolic cages. Data are expressed as the mean ± SEM.
